# Bronchial Carcinoid Tumors with Massive Osseous Metaplasia: A Case Report and Review of the Literature*

**DOI:** 10.5146/tjpath.2018.01457

**Published:** 2020-05-15

**Authors:** Mine Özşen, Ulviye Yalçınkaya, Elif Ülker Akyıldız, Ahmet Sami Bayram, Gökhan Gökalp

**Affiliations:** Department of Pathology, Erzurum Regional Training and Research Hospital, Erzurum, Turkey; Department of Surgical Pathology, Uludag University, Faculty of Medicine, Bursa, Turkey; Department of Thorax Surgery, Uludag University, Faculty of Medicine, Bursa, Turkey; Department of Radiology, Uludag University, Faculty of Medicine, Bursa, Turkey

**Keywords:** Bronchial carcinoid tumor, Carcinoid variants, Osseous metaplasia

## Abstract

Bronchial carcinoid tumors are primary lung neoplasms thought to originate from neuroendocrine cells, i.e. Kulchitsky cells, in the bronchial mucosa, although the type of cellular origin has not been clearly understood. A 61-year-old male patient underwent surgery and microscopic examination of the specimen revealed an anastomosing trabecular bony structure among the nests of tumor cells with round nucleus, granular chromatin, and large eosinophilic cytoplasm. Our case has been deemed worthy of being presented as bronchial carcinoid tumor with exaggerated osseous metaplasia.

## INTRODUCTION

Bronchial carcinoid tumors are primary lung neoplasms thought to originate from neuroendocrine cells, i.e. Kulchitsky cells, in the bronchial mucosa, although the type of cell from which they originate has not been clearly understood. They account for more than 25% of all carcinoid tumors throughout the body, and less than 1% of all such lesions in the lung (1,2).

According to the World Health Organization (WHO) classification in 2015, bronchial carcinoid tumors are classified into typical and atypical tumors based on their histopathological characteristics. Another classification according to the location is as central and peripheral tumors. Furthermore, there are reports that classify such tumors as well-differentiated neuroendocrine carcinoma and moderately-differentiated neuroendocrine carcinoma according to their histopathological characteristics and behavioral potential under the heading of neuroendocrine lung tumors (1,3).

Bronchial carcinoid tumors may present as various morphologic variants. These variants include tumors with metaplastic cartilage and bone growth, tumors containing mucinous stroma, tumors with wide vascular structures, tumors presenting cystic changes, tumors presenting glandular pattern, and tumors containing amyloid-like / sclerotic stroma. Although a carcinoid tumor with metaplastic bone growth is identified as a variant, exaggerated osseous metaplasia is a rare finding in carcinoid tumors (4,5).

## CASE REPORT

**Figure 1:**
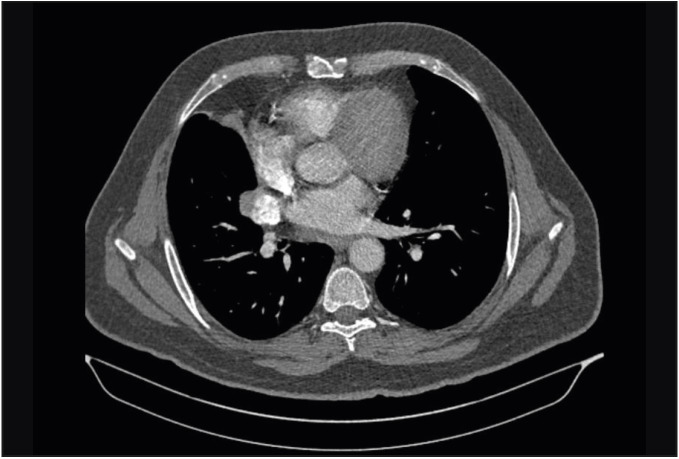
Contrast-enhanced thoracic CT scan; mediastinal window. showing a mass of 37x30 mm in diameter, which results in a complete obstruction in the right middle lobe bronchus, leading to an obstructive atelectasis of the middle lobe.

A 61-year-old male patient presented to an external medical center with the complaint of persistent cough in February 2017 and was referred to our hospital’s clinic of pulmonary diseases for further examination and treatment with the evidence of a pulmonary mass detected during the thoracic computed tomography (CT) examination for etiology. The patient had no active complaints during the admission. The patient was an active smoker for 35 years. There was no specific finding in the patient’s past and family history. The patient initially underwent a chest roentgenogram, followed by a thoracic CT examination. Thoracic CT examination revealed a mass lesion with the dimensions of 4x3 cm containing a significant calcific component in the right hilar region (Figure 1). Fiberoptic bronchoscopy (FOB) was planned for histopathological verification. FOB revealed a complete obstruction of the right middle lobe due to mucosal irregularity, and a punch biopsy was performed.

**Figure 2:**
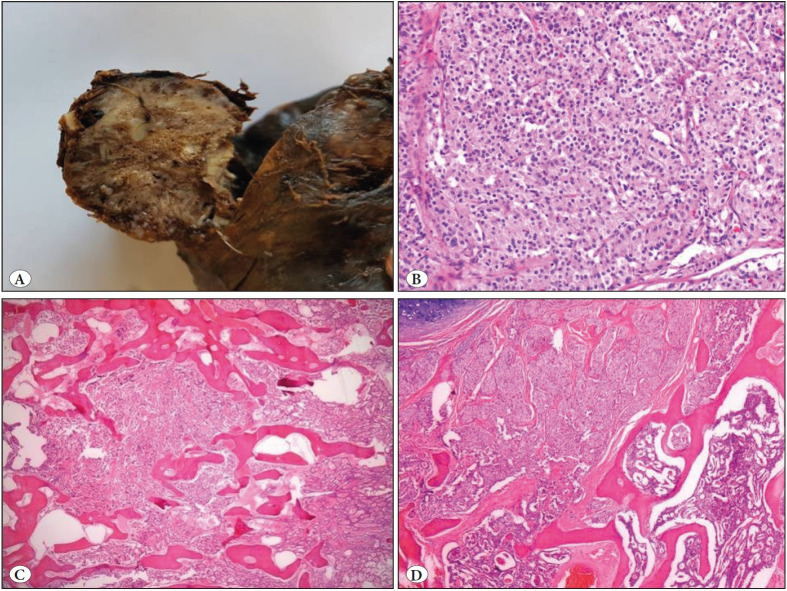
**A)** Macroscopical appearance of a solid, well-circumscribed tumoral lesion in the bronchial lumen and parenchyma, with gray to white cross-sectional areas. **B)** Nests of tumor cells revealing round nucleus, granular chromatin and large eosinophilic cytoplasm (H&E; x400). **C-D)** Nests of tumor cells among anastomosing bone trabeculae (H&E; x100).

A right lower lobectomy was planned since the biopsy specimen was identified as a neuroendocrine tumor. Macroscopical examination of the specimen from the right lower lobectomy revealed a solid, well-circumscribed tumoral lesion in the bronchial lumen and parenchyma, measuring 4x3x3 cm in size, with gray to white cross-sectional areas, and containing locally hard-to-cut regions (Figure 2).

**Figure 3:**
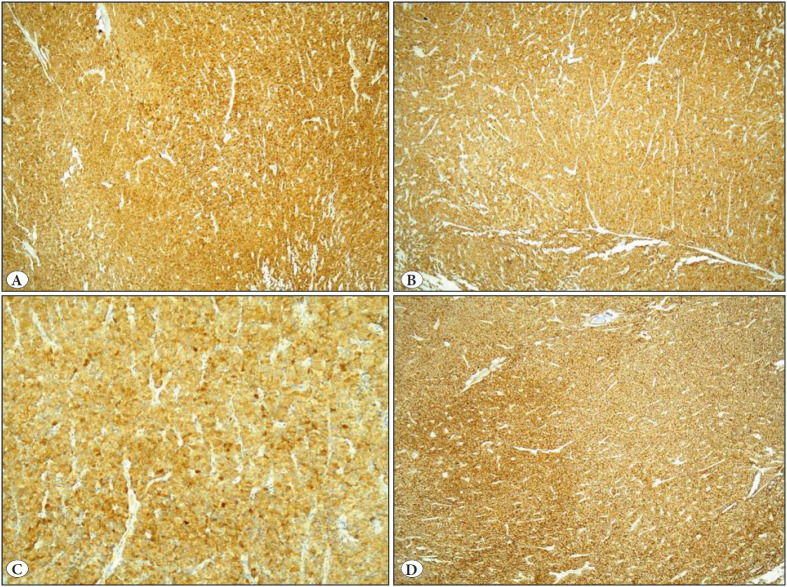
Strong positivity to immunohistochemical staining with **A)** Chromogranin -A (IHC; x100). **B)** Synaptophysin (IHC; x100). **C)** NSE (IHC; x200). **D)** CD56 (IHC; x100).

Histopathological examination of the lesion following decalcification and formalin fixation revealed an anastomosing trabecular bony structure among the nests of tumor cells with round nucleus, granular chromatin, and large eosinophilic cytoplasm (Figure 2). No significant cytological atypia, necrosis or elevated mitotic activity (<2/10 HPF) were observed in tumor cells with normal nucleus-cytoplasm ratio. The cells showed strong positivity with immunohistochemical staining for NSE, CD56, synaptophysin and chromogranin (Figure 3). There were no findings suggestive of metastasis in any of the separate lymph nodes, one from station no:11 and three from the peribronchial region dissected during lobectomy.

Based on histopathological and immunohistochemical examinations, the case was diagnosed as carcinoid tumor with exaggerated osseous metaplasia. The patient has been under clinical follow-up for 17 months and is currently in complete remission following surgery.

## DISCUSSION

Carcinoid tumors are considered as prototypes of neuroendocrine tumors, and were first identified by Langhans in 1867. Gosset and Masson reported in 1914 that these tumors, studied and characterized by various researchers over time, have endocrine properties (6).

The incidence of bronchial carcinoid tumors ranges from 0.1 to 1.5 / 100,000. Such cases are detected at a younger age when compared to the primary lung malignancies (typically under 60 years), and may be seen in a wide range of ages. Bronchial carcinoid tumors are known to be more common in females than in males; however, some recent publications have reported that they have an equal rate of occurrence in females and males, and some even state they are 3.6 times more common in males (1,2,7).

Risk factors for developing bronchial carcinoid tumors include a family history of carcinoid tumors and various genetic mutations. Although it is known that smoking does not play a role in the pathogenesis, a history of smoking is usually present in patients diagnosed with atypical carcinoid tumor (8,9). In our case, the patient had a history of smoking for 35 years and there were no carcinoid tumors in his family history.

Patients with carcinoid tumors usually present with the complaints of dyspnea and hemoptysis since they are endobronchial growing masses. One third of the patients are incidentally detected without presenting any symptoms depending on the involvement of small airways. Paraneoplastic syndromes such as Cushing syndrome and acromegaly can also occur in the cases (10). Our patient presented with the complaint of a long-lasting and persistent cough.

On radiological examinations, such tumors are seen as hilar or perihilar masses; however, they may be detected rarely as peripheral masses as well. While FOB is the preferred imaging modality for centralized tumors, CT is the preferred imaging modality for peripheral tumors. Bronchial carcinoid tumors have a distinctive FOB appearance as a result of their macroscopical features including polypoid form, smooth surface, red to bronze color, and endobronchial growth (11). The diagnosis was made by a biopsy taken during bronchoscopy performed following the detection of a hilar mass on CT.

The WHO classifies carcinoid tumors into two categories according to their histopathological characteristics as typical and atypical carcinoid tumors. The main criteria used in such distinction are mitotic activity and necrosis. In typical carcinoid tumors, which account for 70-90% of all carcinoid tumors and tend to settle in the center, the number of mitoses is less than 2 in 10 HPF’s, with no necrosis. The number of mitosis in atypical carcinoid tumors is between 3 and 9 in 10 HPF’s, and focal necrosis can be detected in such tumors (1,12). No significant cytological atypia, necrosis or elevated mitotic activity (<2/10 HPF) was observed in our case.

In bronchial carcinoid tumors, calcification is present in up to 30% of cases, whereas this number is 10% for ossification. While calcification requires precipitation of calcium salts in sites where prolonged tissue damage occurs due to a number of factors, ossification is a complex process involving osteoblasts and various inducing agents. In carcinoid tumor cells, it is thought that both osteocalcin, defined as an osteogenic differentiation marker, and secretion of bone morphogenetic protein (BMP) that induces differentiation of pluripotent cells into osteoblastic cells have a major role in the ossification in these tumors. Although there are publications reporting that intratumoral ossification in different tumors may be an indicator of metastatic potential, it is currently not possible to establish a relationship between intratumoral ossification and metastatic potential due to the insufficient number of cases of carcinoid tumors (13–17). In our case, there was no evidence suggesting metastasis at the time of diagnosis and after 17 months of follow-up.

When the literature is reviewed, it is noteworthy that cases of carcinoid tumors with exaggerated osseous metaplasia are very rare. The first case was published in 1962, and 23 cases have been reported as case reports so far with no large series (13–15,18–24). In their first relevant series comprising 22 cases published by Cooney et al. in 1979, ossification was found in seven cases, five of which were atypical carcinoid in nature (25). In the series of 63 cases published by Ha et al. in 2013, ossification was found in six cases, all of which were typical carcinoid in nature (26).

We conclude that even the typical carcinoid tumors may present with exaggerated osseous metaplasia, although extremely rare. Complete resection of such tumors will lead to cure without any recurrences or metastasis.

## Conflict of Interest

The authors declare no conflict of interest.

## References

[ref-1] Travis WD, Brambilla E, Burke AP, Marx A, Nicholson AG (2015). World Health Organization Classification of Tumours of Lung, Pleura, Thymus and Heart: Carcinoid Tumor.

[ref-2] Chong Semin, Lee Kyung Soo, Chung Myung Jin, Han Joungho, Kwon O. Jung, Kim Tae Sung (2006). Neuroendocrine tumors of the lung: clinical, pathologic, and imaging findings. Radiographics.

[ref-3] Suster Saul, Moran Cesar A. (2017). Neuroendocrine Carcinoma (Including Small Cell Carcinoma). Diagnostic Pathology: Thoracic.

[ref-4] Tomashefski Joseph F., Cagle Philip T., Farver Carol F., Fraire Armando E. (2008). Dail and Hammar’s Pulmonary Pathology: Volume II: Neoplastic Lung Disease.

[ref-5] Suster S, Moran CA (2015). Biyopsi Yorumları Serisi Akciğerin Biyopsilerinin Yorumu: Karsinoid tümör.

[ref-6] Modlin Irvin M., Lye Kevin D., Kidd Mark (2003). A 5-decade analysis of 13,715 carcinoid tumors. Cancer.

[ref-7] Daddi Niccolò, Schiavon Marco, Filosso Pier Luigi, Cardillo Giuseppe, Ambrogi Marcello Carlo, De Palma Angela, Luzzi Luca, Bandiera Alessandro, Casali Christian, Ruffato Alberto, De Angelis Verena, Andriolo Luigi Gaetano, Guerrera Francesco, Carleo Francesco, Davini Federico, Urbani Moira, Mattioli Sandro, Morandi Uliano, Zannini Piero, Gotti Giuseppe, Loizzi Michele, Puma Francesco, Mussi Alfredo, Ricci Alberto, Oliaro Alberto, Rea Federico, Multi-Institutional Italian Pathology Group (2014). Prognostic factors in a multicentre study of 247 atypical pulmonary carcinoids. Eur J Cardiothorac Surg.

[ref-8] Kaifi Jussuf T., Kayser Gian, Ruf Juri, Passlick Bernward (2015). The Diagnosis and Treatment of Bronchopulmonary Carcinoid. Dtsch Arztebl Int.

[ref-9] Bertino Erin M., Confer Patricia D., Colonna Jorge E., Ross Patrick, Otterson Gregory A. (2009). Pulmonary neuroendocrine/carcinoid tumors: a review article. Cancer.

[ref-10] Detterbeck Frank C. (2010). Management of carcinoid tumors. Ann Thorac Surg.

[ref-11] Jeung Mi-Young, Gasser Bernard, Gangi Afshin, Charneau Dominique, Ducroq Xavier, Kessler Romain, Quoix Elisabeth, Roy Catherine (2002). Bronchial carcinoid tumors of the thorax: spectrum of radiologic findings. Radiographics.

[ref-12] Cagle PT, Allen TC (2016). Lung and pleural pathology ebook.

[ref-13] Shin M. S., Berland L. L., Myers J. L., Clary G., Zorn G. L. (1989). CT demonstration of an ossifying bronchial carcinoid simulating broncholithiasis. AJR Am J Roentgenol.

[ref-14] Osmond Allison, Filter Emily, Joseph Mariamma, Inculet Richard, Kwan Keith, McCormack David (2016). Endobronchial Carcinoid Tumour with Extensive Ossification: An Unusual Case Presentation. Case Rep Med.

[ref-15] Khalil M, Eltorky M (2013). Bronchial Carcinoid with massive ossification: A case report and review of literature. Int J Clin Exp Pathol.

[ref-16] Hadano Atsuko, Hirabayashi Kenichi, Yamamuro Hiroshi, Takanashi Yumi, Yamada Misuzu, Kawanishi Aya, Kawaguchi Yoshiaki, Furukawa Daisuke, Nakagohri Toshio, Imai Yutaka, Nakamura Naoya, Mine Tetsuya (2016). Bone morphogenetic protein-2 expression in an intraductal papillary mucinous neoplasm with marked ossification: A case report. Pathol Int.

[ref-17] Kim Gou Young, Kim Jhingook, Kim Tae Sung, Han Joungho (2009). Pulmonary adenocarcinoma with heterotopic ossification. J Korean Med Sci.

[ref-18] Vanmaele L., Noppen M., Frecourt N., Impens N., Welch B., Schandevijl W. (1990). Atypical ossification in bronchial carcinoid. Eur Respir J.

[ref-19] Personne C., Toty L., Constantinescu-Wappler C., Hertzog P., Audebaud G., Guilloux M., Juteau C. (1972). Ossified bronchial carcinoid tumor. Rev Tuberc Pneumol (Paris).

[ref-20] Llombart A Jr, Gomar GF (1962). Ossifying argentaffin bronchial carcinoid and acromegaly. Rev Clin Esp.

[ref-21] Griese M., Reinhardt D., Reifenhäuser A., Irlich G. (1987). Chronic recurrent pneumonias in ossifying bronchial carcinoid tumor. Monatsschr Kinderheilkd.

[ref-22] Tsubochi Hiroyoshi, Endo Shunsuke, Oda Yoshinao, Dobashi Yoh (2013). Carcinoid tumor of the lung with massive ossification: report of a case showing the evidence of osteomimicry and review of the literature. Int J Clin Exp Pathol.

[ref-23] Troupin Rosalind H. (1968). Ossifying bronchial carcinoid: A case report. American Journal of Roentgenology.

[ref-24] Bürrig KF, Frenzel H (1985). Ossifying bronchial carcinoid in childhood: Report of a clinical case. Pathologe.

[ref-25] Cooney T., Sweeney E. C., Luke D. (1979). Pulmonary carcinoid tumours: a comparative regional study. J Clin Pathol.

[ref-26] Ha Sang Yun, Lee Jae Jun, Cho Junhun, Hyeon Jiyeon, Han Joungho, Kim Hong Kwan (2013). Lung parenchymal invasion in pulmonary carcinoid tumor: an important histologic feature suggesting the diagnosis of atypical carcinoid and poor prognosis. Lung Cancer.

